# Antiproliferative and Apoptotic Activity of *Chamaecyparis obtusa* Leaf Extract against the HCT116 Human Colorectal Cancer Cell Line and Investigation of the Bioactive Compound by Gas Chromatography-Mass Spectrometry-Based Metabolomics

**DOI:** 10.3390/molecules201018066

**Published:** 2015-10-02

**Authors:** Hye-Youn Kim, Seul-Gi Lee, Taek-Joo Oh, Sa Rang Lim, So-Hyun Kim, Hong Jin Lee, Young-Suk Kim, Hyung-Kyoon Choi

**Affiliations:** 1 College of Pharmacy, Chung-Ang University, Seoul 156-756, Korea; E-Mails: khyoun412@naver.com (H.-Y.K.); www_12@hanmail.net (S.-G.L.); naya8729@naver.com (T.-J.O.); lsr911210@gmail.com (S.R.L.); sohyunvision@gmail.com (S.-H.K.); 2 Department of Food Science and Technology, Chung-Ang University, Anseong 456-756, Korea; E-Mail: hongjin@cau.ac.kr; 3 Department of Food Science and Technology, Ewha Womans University, Seoul 120-750, Korea; E-Mail: yskim10@mm.ewha.ac.kr

**Keywords:** *Chamaecyparis obtusa*, human colorectal cancer, metabolite profiling, gas chromatography-mass spectrometry, anthricin

## Abstract

*Chamaecyparis obtusa* (CO) belongs to the Cupressaceae family, and it is found widely distributed in Japan and Korea. In this study, the anti-proliferative activities of the methanol and water extracts of CO leaves against a human colorectal cancer cell line (HCT116) were investigated. The methanol extract of CO leaves, at a concentration of 1.25 µg/mL, exhibited anti-proliferative activity against HCT116 cells, while displaying no cytotoxicity against Chang liver cells. Comparative global metabolite profiling was performed using gas chromatography-mass spectrometry coupled with multivariate statistical analysis, and it was revealed that anthricin was the major compound contributing to the anti-proliferative activity. The activation of c-Jun *N*-terminal kinases played a key role in the apoptotic effect of the methanol extract of CO leaves in HCT116 human colon cancer cells. These results suggest that the methanol extract and anthricin derived from CO leaves might be useful in the development of medicines with anti-colorectal cancer activity.

## 1. Introduction

*Chamaecyparis obtusa* (CO), commonly known as *hinoki*, belongs to the Cupressaceae family, and it is grown in China, Japan, and the Republic of Korea [1,2,3]. CO lumber has been used widely in the manufacture of bathtubs, furniture, and pillow stuffing material, and as a source of CO oil. The CO traditionally used in the treatment of urinary tract infections [4,5], and it considered as natural sources of medicine because of its various bioactivities. The strong antimicrobial activity of CO leaf extracts and the acaricidal activity of a CO leaf-derived compound have been reported in previous studies [6,7]. In addition, essential oils derived from CO are known to have anti-inflammatory activities [8]. The neuroprotective effects of bioflavonoid derived from CO leaves have also been reported recently [9]. Various kinds of monoterpenoids, sesquiterpenoids, flavonoids, and bioflavonoids are known to contribute to the various bioactivities of CO leaves [8,9,10,11,12,13]. However, to the best of our knowledge, there is no report on the anti-proliferative activity of the CO leaf extract against human colorectal cancer cell lines and on the compounds contributing to this effect.

Metabolomics is defined as the identification and quantification of all the metabolites present in a biological system under a given set of conditions. Metabolomics, together with genomics, transcriptomics, and proteomics can be applied in the study of bioactive compounds contained in various natural resources [14]. The major analytical platforms in metabolomics include nuclear magnetic resonance spectrometry (NMR), liquid chromatography-mass spectrometry, and gas chromatography-mass spectrometry (GC-MS) [15]. However, only one metabolomic approach (NMR-based) has been utilized recently with the Cupressaceae family for the metabolic characterization of seasonal changes in *Juniperus communis* berry extracts [16].

The hypothesis of this study was that CO leaves might have an antiproliferative activity against the HCT116 cell line. Therefore, the main aims of this experiment were to investigate the anti-proliferative activity of CO leaf extracts and identify the specific compounds contributing to this effect by using GC-MS coupled with multivariate statistical analysis.

## 2. Results and Discussion

### 2.1. Antiproliferative Activity of Various Extracts

**Figure 1:**
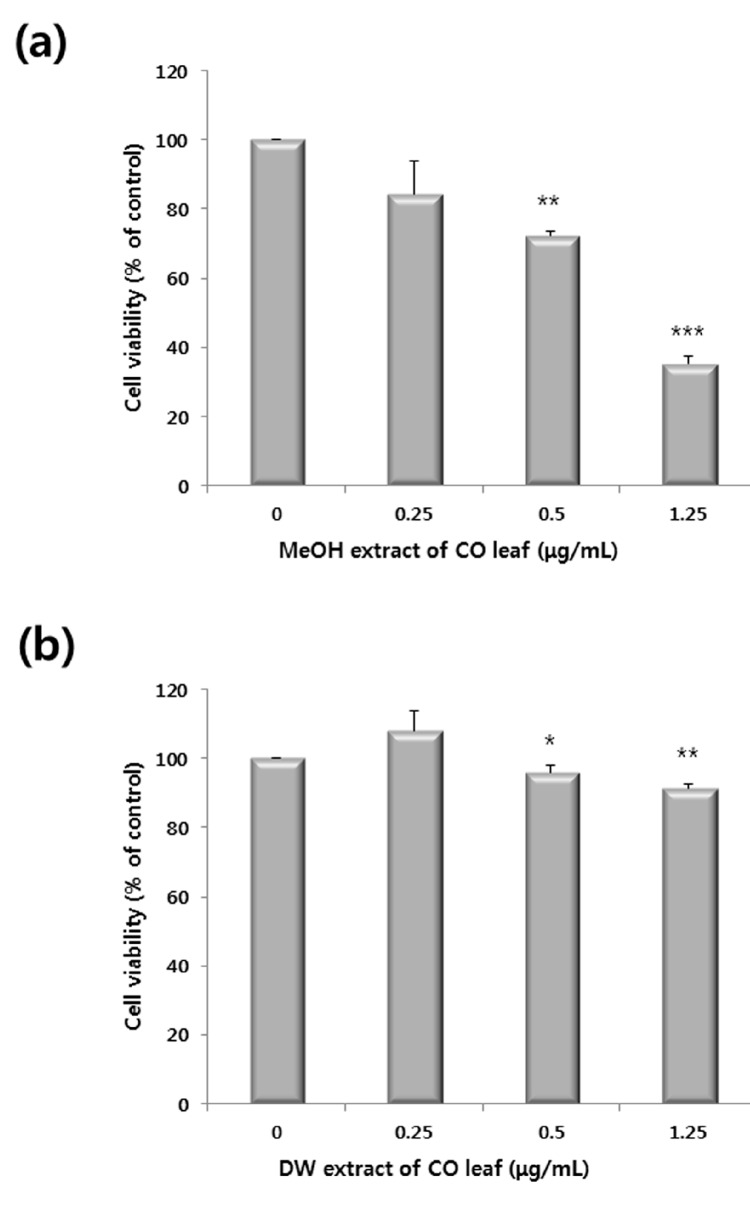
Antiproliferative activity of (**a**) methanol extracts and (**b**) water extracts of *Chamaecyparis obtusa* (CO) leaves (0–1.25 μg/mL) against HCT116 human colon cancer cells. Cells were treated with the extracts for 24 h. Data represent the mean of the percentage of control in triplicate tests. * *p* < 0.05, ** *p* < 0.01 and *** *p* < 0.001 indicate statistically significant difference compared to the control analyzed by the student’s *t*-test.

Figure 1 shows the anti-proliferative activity methanol and water extracts of CO leaves. The methanol extract, at concentrations above 1.25 µg/mL, showed anti-proliferative activities against HCT116 cells, whereas the water extract did not elicit marked activities because of the cell viability being greater than 90%.

**Figure 2:**
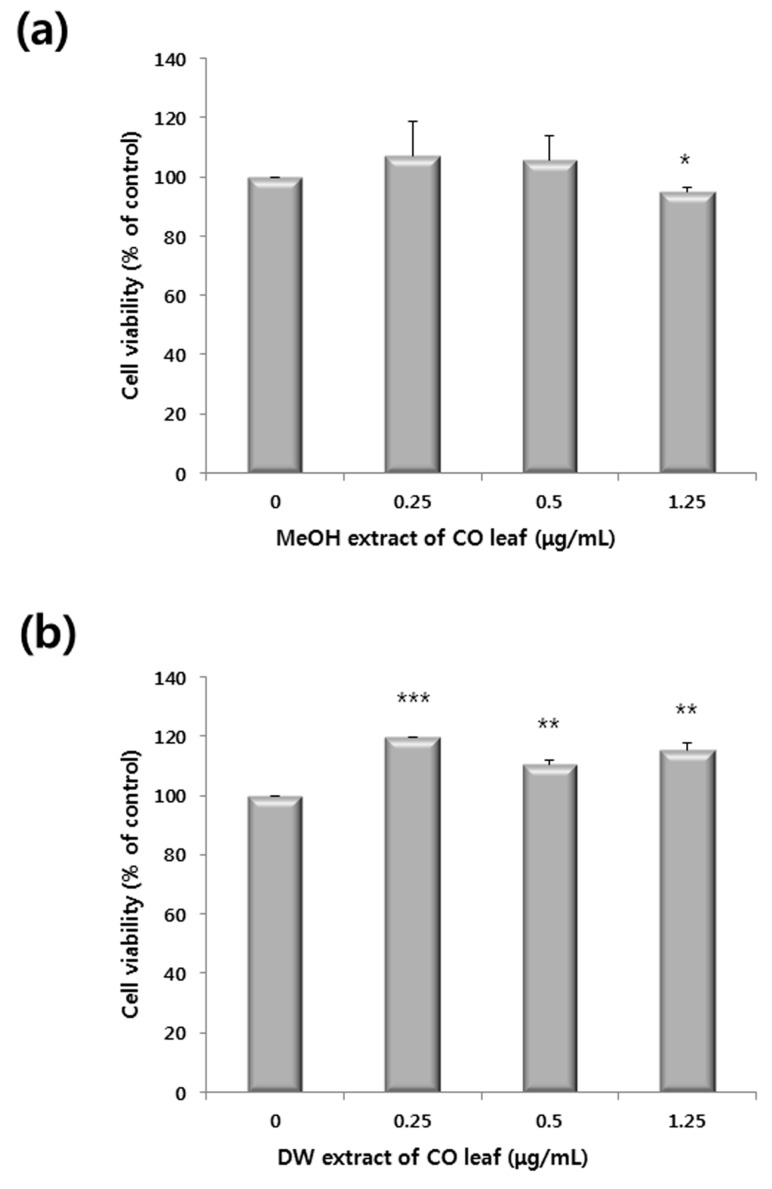
Cell viability of (**a**) methanol extracts and (**b**) water extracts of *Chamaecyparis obtusa* (CO) leaves (0–1.25 μg/mL) against Chang liver cells. Cells were treated with the extracts for 24 h. Data represent the mean of the percentage of control in triplicate tests. * *p* < 0.05, ** *p* < 0.01 and *** *p* < 0.001 indicate statistically significant difference compared to the control analyzed by the student’s t-test.

The cell viabilities of the methanol and water extracts against Chang liver cells are shown in Figure 2. Although the methanol extract significantly decreased the cell viability at 1.25 μg/mL, the cell viability remained above 90%. Therefore, MeOH extract of CO leaves demonstrated selective cytotoxicity against HCT116 human colon cancer cells, while it was ineffective in Chang liver cells. As described above, we observed antiproliferative activity with 1.25 µg/mL of methanol extract. Hence, this concentration of methanol extract was chosen for further studies.

It has previously been reported that epigallocatechin gallate and epigallocatechin showed antiproliferative and apoptotic activity against the HCT116 cell line at concentrations of 50–200 µM [17]. Grape seed extract also showed anticancer activity against these cells at concentrations of 25–100 µg/mL [18]. *Artemisia herba* has also been reported to show antiproliferative activity against the HCT116 cell line at concentrations of 25–200 µg/mL [19]. Additionally, among the members of the Cupressaceae family, ethanol extract of *Juniperus phoenicea* L. showed antiproliferative activity against HCT116 cells at concentrations of 5–50 µg/mL [20]. Hinokitiol, isolated from *Thujopsis dolabrata*, also has potential apoptotic activity against HCT116 colon cancer cells and SW620 cells at concentrations of 5–10 µM [21]. The antiproliferative effects of widdrol, from *Juniperus chinensis*, against the human colon adenocarcinoma cell line HT29, were also observed at concentrations of 16–64 µg/mL [22]. Considering these previously published results, the methanol extract of CO leaves could represent a potent bioresource for the development of medicines with anti-colon cancer activity.

### 2.2. Comparative Global Metabolite Profiling of Methanol and Water Extracts Using GC-MS

**Figure 3:**
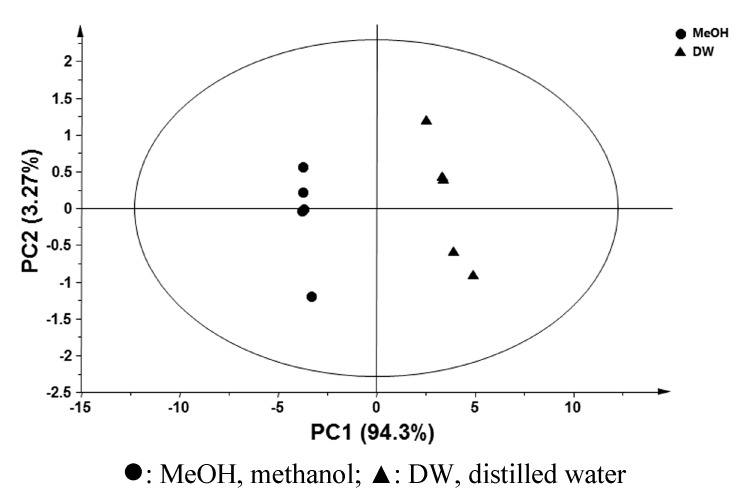
Principal component analysis (PCA)-derived score plots of methanol and water extracts of *Chamaecyparis obtusa* leaves using five biological replicates.

PCA was performed to compare the metabolic profiles in methanol and water extracts of CO leaves. As shown in Figure 3, there was a separation between methanol and water extracts of CO in the PCA-derived score plots mainly by principal component 1. The results imply that the metabolic profiles in methanol and water extracts of CO leaves varied according to the extraction solvents.

Comprehensive metabolic profiling of CO leaf extracts was performed using GC-MS to investigate the major compounds contributing to the anti-proliferative activity against HCT116 cells. As described in Table 1, 16 metabolites in total were identified in the methanol and water extracts of CO leaves, and anthricin was identified in the methanol extract of CO leaf. The anthricin (deoxypodophyllotoxin) has been found in the dried root of *Anthriscus sylvestris*, which is a wild plant from Europe, North America, Africa, and Asia [23]. Anthricin has also been found in *Pulsatilla koreana* [24]. Anthricin has shown anticancer activity against leukemia, and prostate, breast, and uterine cervical tumors [25,26,27,28,29,30]. However, there have been no previous reports regarding the existence of anthricin in CO.

**Table 1:** Relative intensity of various metabolites in the methanol (MeOH) and distilled water (DW) extracts of *Chamaecyparis obtusa* leaves. All data are presented as mean ± standard error of the mean of five replicates. * *p* < 0.05 (student’s *t*-test); ** *p* < 0.01 (Student’s *t*-test). RT, retention time; ND, not detected.

Compound	RT (min)	Relative Intensity	
		DW	MeOH
**Alcohols**			
Glycerol *	10.23	1.90 ± 0.23	1.19 ± 0.12
Mannitol *	28.56	0.51 ± 0.07	0.28 ± 0.04
Myo-inositol *	33.37	2.16 ± 0.23	1.12 ± 0.11
**Amino acids**			
Aspartic acid **	16.58	0.06 ± 0.01	ND
Glutamic acid **	19.30	0.14 ± 0.02	ND
Serine **	12.47	0.05 ± 0.00	ND
**Fatty acids**			
Palmitic acid **	32.25	ND	0.09 ± 0.01
**Lignan**			
Anthricin **	57.14	ND	0.21 ± 0.02
**Organic acids**			
Succinic acid *	11.27	0.13 ± 0.02	0.08 ± 0.01
Malic acid **	15.80	4.61 ± 0.51	0.76 ± 0.08
Xylonic acid **	22.53	1.05 ± 0.10	0.25 ± 0.03
**Phenolic acids**			
Shikimic acid *	25.21	9.28 ± 1.03	5.73 ± 0.54
**Sterol**			
β-Sitosterol **	57.92	ND	0.33 ± 0.03
**Sesquiterpenes**			
Thujopsene **	14.44	ND	0.06 ± 0.01
β-Eudesmol **	22.64, 22.88	ND	4.03 ± 0.46
**Sugar**			
Glucose *	30.44	40.36 ± 11.01	20.15 ± 2.41

**Figure 4:**
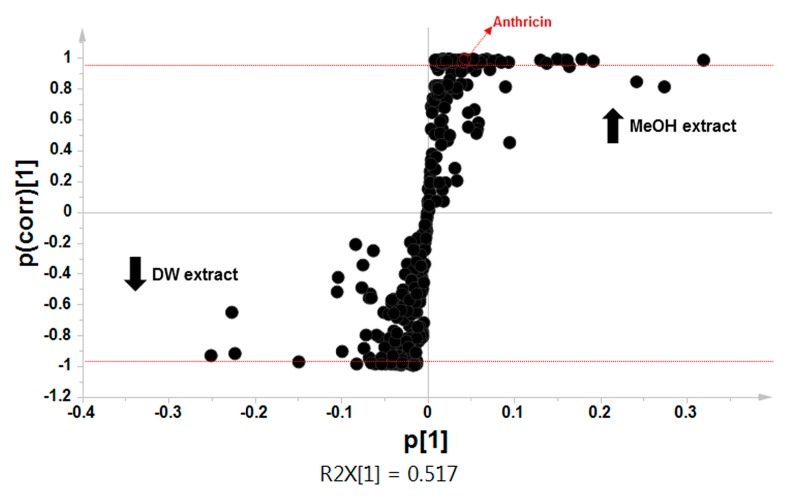
S-plots obtained by orthogonal partial least-squares discriminant analysis (OPLS-DA) of methanol and water extracts of *Chamaecyparis obtusa* leaves.

OPLS-DA was performed to obtain clearer separation of the two extracts and to reveal major compound contributing to the antiproliferative activity of CO leaf extracts. In Figure 4, the S-plot clearly shows the separation of predictive variables between the methanol and water extracts. The S-plot visualizes the separation of various variables contributing to group separation. We observed the statistically significant metabolites from the methanol and water extracts using OPLS-DA (Table 2). Various metabolites, such as alcohols, organic acids, sugar, and phenolic acid were identified in the two extracts, however, palmitic acid, thujopsene, anthricin, β-eudesmol and β-sitosterol were relatively higher in the methanol extract than in the water extract (*p* < 0.01).

**Table 2:** Metabolites identified in methanol (MeOH) and distilled water (DW) extracts of *Chamaecyparis obtusa* leaves separated by orthogonal partial least-squares discriminant analysis (OPLS-DA).

MeOH Extract (*p* < 0.01)	DW Extract (*p* < 0.05)
Palmitic acid	Glycerol
Thujopsene	Mannitol
Anthricin	Myo-inositol
β-Sitosterol	Aspartic acid
β-Eudesmol	Glutamic acid
	Serine
	Succinic acid
	Malic acid
	Xylonic acid
	Shikimic acid

**Figure 5:**
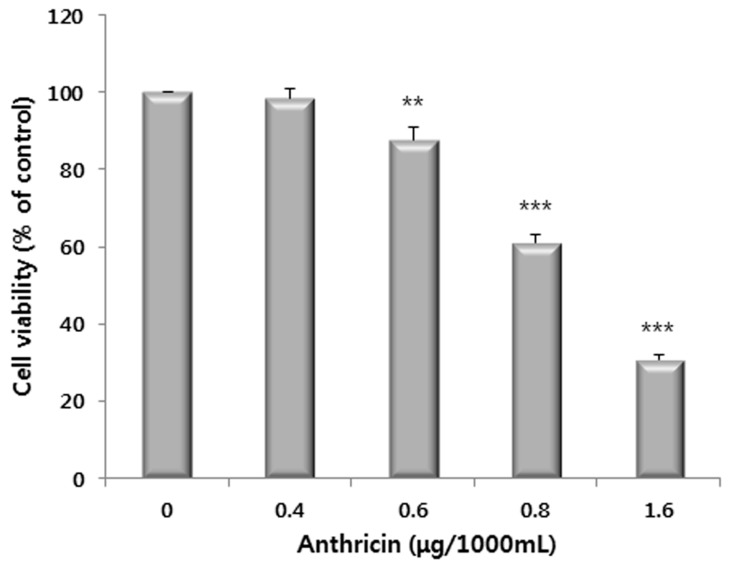
Effects of anthricin on cell proliferation in HCT116 human colon cancer cells. Cells were treated with anthricin (0–1.6 μg/1000 mL) for 24 h. Data represent the mean of the percentage of control in triplicate tests. ** *p* < 0.01 and *** *p* < 0.001 indicate statistically significant difference compared to the control analyzed by the student’s *t*-test.

We aimed to confirm if the anthricin contained in the methanol extracts of CO leaves has antiproliferative activity against HCT116 cells. We determined the amount of anthricin in the methanol extract of CO leaves as 36.1 μg/1000 mL (Table 3). Based on the study, we investigated the antiproliferative activity of anthricin standard compound of various concentrations. As shown in Figure 5, although the concentration of standard anthricin (1.6 μg/1000 mL) was 1/25 concentration of the anthricin content in methanol extract, the single anthricin standard showed similar antiproliferative activity of anthricin contained in methanol extract. Previous studies have also shown that crude extracts exhibit lower activity than sub-fractions, isolated compounds, or standards. The cranberry extracts showed lower activity than purified cyanidin glycosides in free radical scavenging activity, and the extracts of *Platycodon grandiflorum* had a lower cytotoxicity on human cancer cells (HT-29, HRT-18 and HepG2) than the other sub-fractions and isolated compounds [31,32]. Thus, our result is in accordance with those previous reports, and we could speculate that various metabolites contained in the methanol extract might lower the antiproliferative activity of anthricin.

**Table 3:** The regression equation and *R*^2^ value obtained from various concentration of anthricin, and the content of anthricin in the methanol extracts of *C. obtusa* leaves.

Compound	Regression Equation	*R*^2^ Value	Absolute Concentration ^a^ (μg/1000 mL)
Anthricin	y = 0.0029x − 0.6787	0.9945	36.1

^a^ Anthricin concentration contained in methanol extract (1.25 μg dried extract/mL) of *C. obtusa* leaves.

### 2.3. Western Blot Analysis

Expressions of the apoptosis-related enzymes, caspase-3 and PARP (poly ADP ribose polymerase) were investigated using western blot analysis after exposure of cells to the methanol extract of CO leaves for 6 h to investigate the mechanisms underlying the cytotoxic effects of the extract. Caspase-3 is known as important mediator leading to the proteolytic cleavage of PARP, and causing apoptotic cell death [33,34].

**Figure 6:**
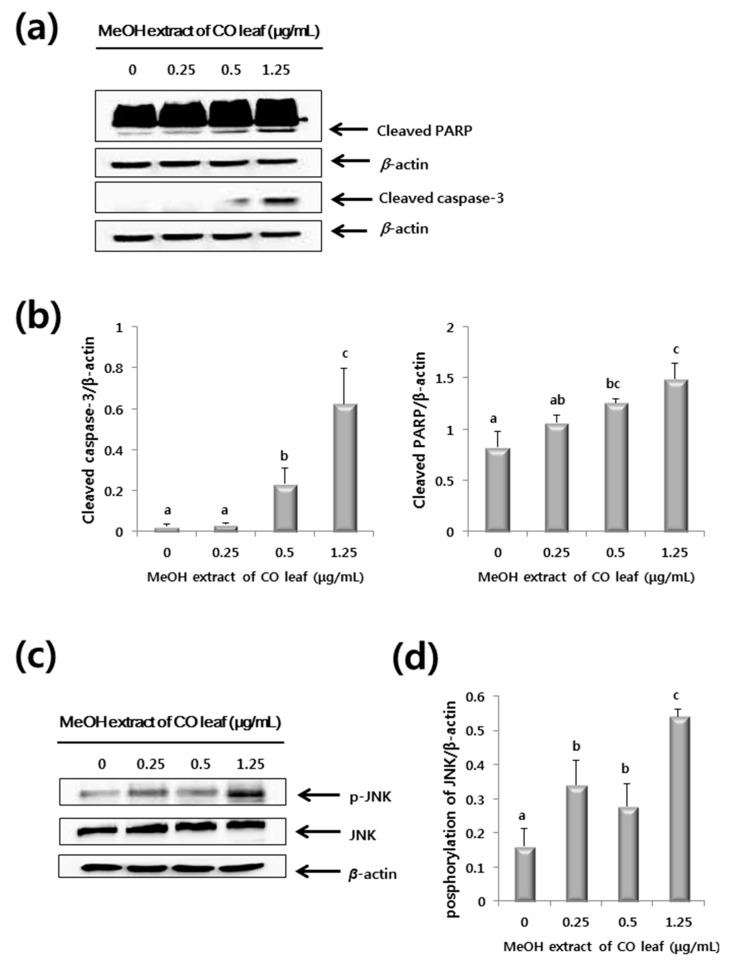
Western blot of HCT116 cells treated with methanol extracts of *Chamaecyparis obtusa* leaves. (**a**) Representative western blot of caspase-3 and poly ADP ribose polymerase (PARP) protein expression; (**b**) Expression levels of caspase-3 and PARP protein. The level of each protein was normalized to beta-actin protein levels; (**c**) A representative western blot of phosphorylated-c-Jun *N*-terminal kinases (p-JNK) protein expression; (**d**) Expression levels of p-JNK protein. The results are from three independent experiments and presented as mean ± standard deviation. The letter labels on the graph indicate statistically significant differences between samples (*p* < 0.05) based on one-way ANOVA with Tukey’s *post hoc* test.

As shown in Figure 6a,b, the relative levels of cleaved caspase-3 and cleaved PARP were increased by methanol extract of CO leaves in dose-dependent manner. These results suggest that methanol extract of CO leaves might cause apoptotic cell death via a caspase-dependent pathway.

**Figure 7:**
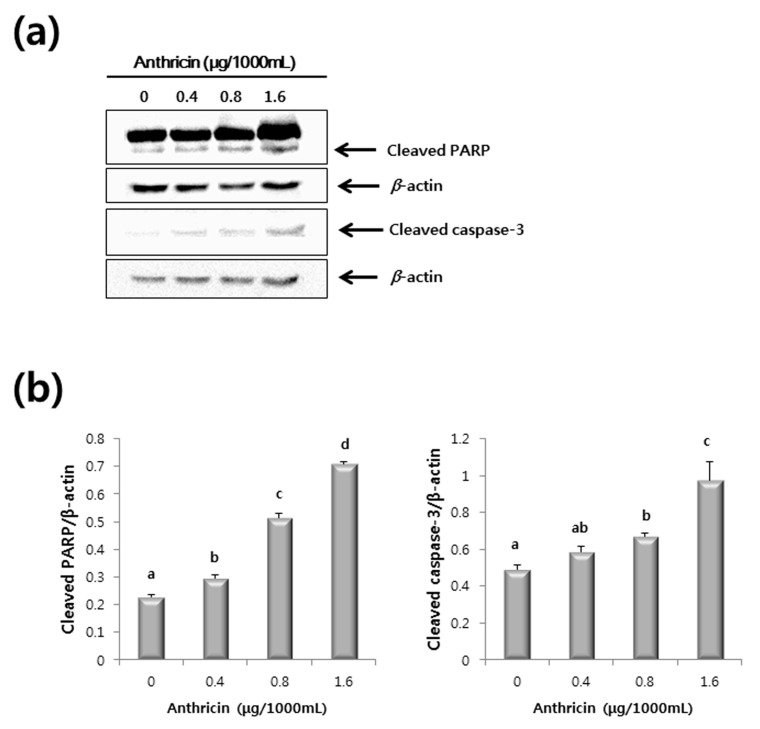
Western blot of HCT116 cells treated with anthricin (0–1.6 μg/1000mL). (**a**) Representative western blot of caspase-3 and poly ADP ribose polymerase (PARP) protein expression; (**b**) Expression levels of caspase-3 and PARP protein. The level of each protein was normalized to beta-actin protein levels. The letter labels on the graph indicate statistically significant differences between samples (*p* < 0.05) based on one-way ANOVA with Tukey’s *post hoc* test.

In addition, as shown in Figure 7a,b, similar results were obtained in experiments performed with anthricin standard compound. Treatment of HCT116 cells with anthricin (0–1.6 μg/1000mL) caused significant increases in the levels of cleaved caspase-3 and cleaved PARP expression. These data indicate that the anthricin contained in the methanol extract of CO leaves induces apoptosis of HCT116 human colorectal cancer cell line.

**Figure 8:**
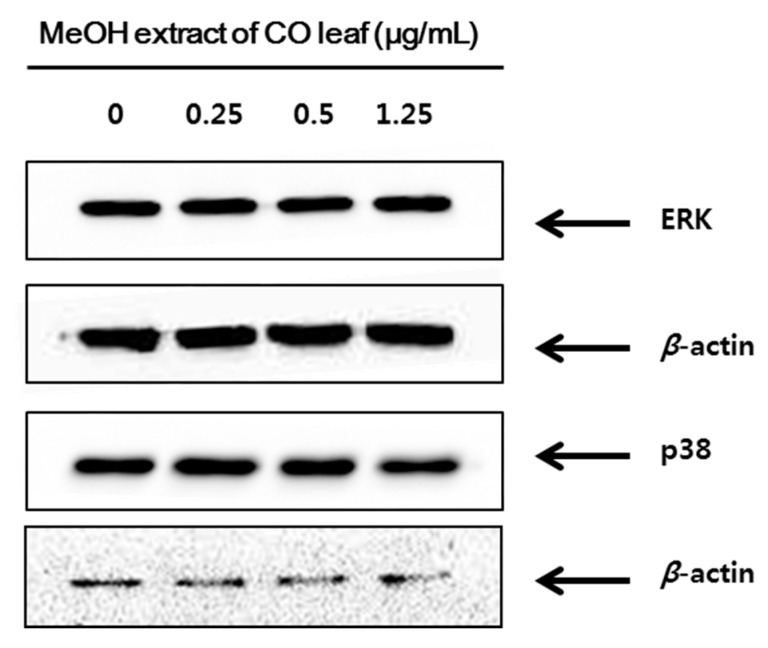
Western blot of HCT116 cells treated with methanol extracts of *C. obtusa* leaves. Representative western blot of ERK and p38 protein expression.

Mitogen-activated protein kinase (MAPK) was known to be involved in cell proliferation, differentiation, apoptosis, and signaling cascades caused by external stimuli associated with tumor invasion and metastasis [35,36,37]. MAPK includes three major enzymes: extracellular signal-regulated kinase (ERK), c-Jun *N*-terminal kinase (JNK), and p38 mitogen-activated protein kinase (p38 MAPK) [38]. The expressions of JNK, ERK and p38 were analyzed by western blot analysis to investigate the contribution of those to apoptotic cell death of HCT116 by methanol extract of CO leaves. As shown in Figure 6c,d, the level of the phosphorylated JNK (p-JNK) was increased in HCT116 cells by the treatment of methanol extract of CO leaves in dose-dependent manner. However, there were no increased phosphorylation of ERK and p38 (Figure 8).

There have been several reports showing that the anti-proliferative activity of various agents such as resveratrol, butyrate, phenethyl isothiocyanate, and curcumin (diferuloylmethane) against colorectal cancer cell lines occurs through the JNK pathway, but not the ERK or p38 pathways [39,40,41,42]. As a stress-activated protein kinase, JNK phosphorylates c-Jun on Ser 63 and Ser 73 residues, and numerous studies indicate that JNK has a critical role in regulating cellular apoptosis factors [43,44]. JNK mediates the release of cytochrome c from mitochondria by modulating the activity of pro-apoptotic and anti-apoptotic mitochondrial proteins, such as the Bcl-2 family, and activates the caspase signaling cascade [45,46,47,48,49,50].

The level of p-JNK increased in response to treatment with the methanol extract of CO leaves, and it was presumed that this might cause mitochondrial dysfunction. Thereafter, the elevation of cleaved caspase-3 levels through cytochrome C activation induced the activation of PARP, which then lead to the apoptotic death of the HCT116 cells. Taken together, this study suggests that activation of p-JNK is a key event in the regulation of the apoptotic effect of the methanol extract of CO leaves on HCT116 cells. Our data provides evidence that the CO leaf extract might be an alternative anti-cancer agent that modulates the phosphorylation of JNK and induces apoptosis in human colon cancer cells.

## 3. Experimental Section

### 3.1. Sample Preparation

CO leaves were collected from the Jeonnam Forest Resource Research Institute, which is located in Na-ju, the southwestern part of the Republic of Korea on 12 September 2012. This study also did not involve any endangered or protected species. The collected leaves were freeze-dried (FDU-1200, EYELA, Miyagi, Japan) and powdered in a grinder. The freeze-dried leaves were extracted using a microwave-assisted extraction system (MARSX, CEM Corporation, NC, USA). Briefly, 3 g of freeze-dried leaf powder was suspended in 30 mL of methanol and distilled water (DW) in a 100-mL Teflon PFA (perfluoroalkoxy)-lined extraction vessel. The vessels were placed into the microwave apparatus and subjected to microwave irradiation (400 W) at 80 °C for 10 min. After microwave extraction, the mixture in each vessel was allowed to cool down at room temperature for 10 min. The supernatants were then collected and filtered through filter paper (Whatman No. 4, Whatman, Kent, UK). The extracted samples were stored at −80 °C until further analysis.

### 3.2. Antiproliferative Activity against the HCT116 Cell Line and Viability Test Using Chang Liver Cell Line

HCT116 cells and Chang liver cells (American Type Culture Collection, Manassas, VA, USA) were cultured in Dulbecco’s modified Eagle’s medium (DMEM) supplemented with 10% fetal bovine serum (Hyclone Labs, Logan, UT, USA) and 1% antibiotics (penicillin and streptomycin) at 37 °C in a humidified incubator with 5% CO_2_. Each sample (100,000 µg/mL) of methanol and water extract was dissolved in dimethylsulfoxide (DMSO) and filtered with 0.45 µm syringe filters (polytetrafluoroethylene). The stock sample (10 mM) of anthricin standard (deoxypodophyllotoxin; 95%, Natural Products Bank at the Institute for Korea Traditional Medical Industry, Gyeongbuk, Korea) was dissolved in DMSO. Three concentrations (1.25, 0.5, and 0.25 µg/mL) of each extract sample and four concentrations (1.6, 0.8, 0.6, and 0.4 µg/1000 mL) of anthricin standard were diluted in DMEM. Cell cytotoxicity and viability were determined using a 3-(4,4-dimethylthiazol-2-yl)-2,5-diphenyltetrazolium bromide (MTT) (Sigma-Aldrich, St. Louis, MO, USA) assay. The cells were plated out into 96-well plates at a density of 2 × 10^5^ cells/mL. Cells were cultured overnight for preconditioning, the culture medium was aspirated, and the cells were exposed to different concentrations of CO leaf extract for 24 h. Thereafter, 10 μL of MTT (Sigma-Aldrich, St. Louis, MO, USA) in PBS was added to each well and incubated for 1 h at 37 °C, after which the formazan crystals that formed were dissolved in 100 μL of DMSO. The optical density (OD) was recorded at 570 nm on a microplate spectrophotometer (xMark, Bio-Rad, Berkeley, CA, USA).

### 3.3. Global Metabolite Profiling Using GC-MS

To analyze the metabolites in the extracted samples, a trimethylsilylation (TMS) derivatization reaction was performed on the samples. In brief, 100 μL of each sample solution was transferred into a GC vial and then dried with nitrogen gas flow. Subsequently, 30 µL of 200 µg/mL methoxylamine hydrochloride in pyridine, 50 μL of *N*,*O*-Bis (trimethylsilyl) trifluoroacetamide (BSTFA; Alfa Aesar, Ward Hill, MA, USA) containing 1% trimethyl chlorosilane (TMCS), and 10 µL of 2-chloronaphthalene (Tokyo Chemical Industry Co., Ltd., Tokyo, Japan; 250 mg/mL in pyridine as an internal standard) were added to the dried vials. The derivatized samples were incubated for 60 min at 60 °C before GC-MS analysis.

GC-MS analysis was performed using a 7890A Agilent GC (Agilent Technologies, Santa Clara, CA, USA) model equipped with an autosampler (7683 B series, Agilent Technologies), and a 5975C mass selective detector (Agilent Technologies) system. A DB-5 ms column (30 m length × 0.25 mm internal diameter × 0.25 μm film thickness) was used with a constant flow of 1.0 mL/min of helium as carrier gas. Chemstation software (Agilent Technologies) was used to process the chromatographic and mass spectrometric data. The initial oven temperature was set at 70 °C and then increased to 150 °C (at 5 °C/min). The temperature was then increased to 250 °C (at 3 °C/min; hold 2 min) and finally to 320 °C (at 15 °C/min; hold 3 min). Each extracted sample was injected using the split mode at a split/splitless ratio of 1:10. The electron impact mode with ionization energy of 70 eV was used for mass data collection. The mass range was 50–600 Da, and the data were collected in full scan mode. The resulting GC-MS profile was analyzed by matching the chromatogram of the NIST-Wiley Mass spectra Library with a commercially available standard. An automated mass spectral deconvolution and identification system; AMDIS [51] for mass spectral deconvolution was used for processing multiple datasets. All GC-MS datasets were normalized using the internal standard. The relative levels of each metabolite were obtained by dividing the percentage area of each metabolite by the percentage area of the internal standard.

### 3.4. Quantification of Anthricin by GC-MS

**Figure 9:**
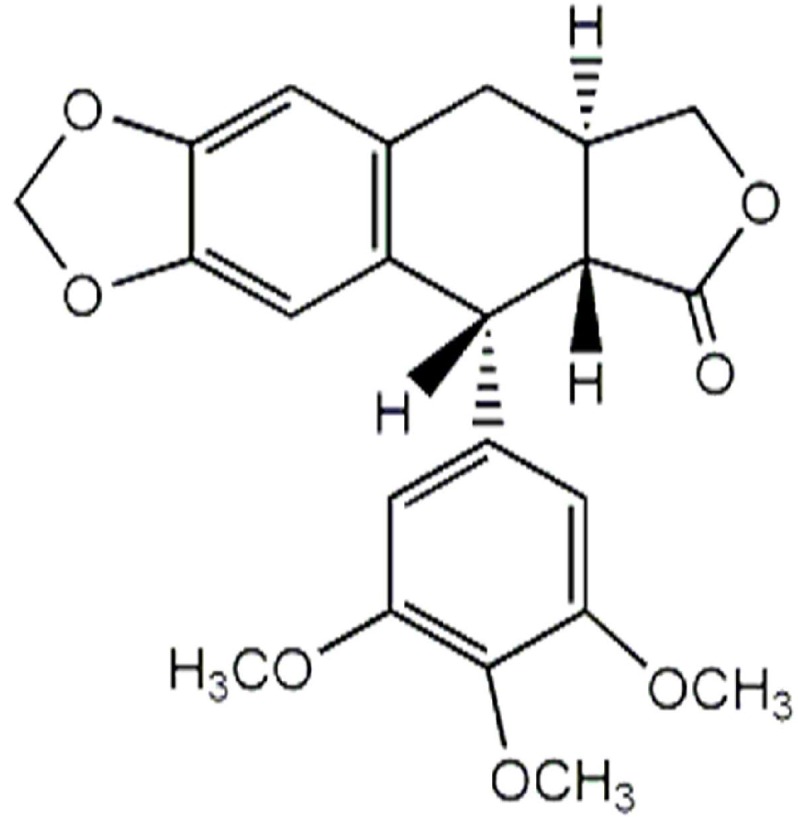
The chemical structure of anthricin (deoxypodophyllotoxin).

Quantitative analysis of anthricin (Figure 9) was carried out with GC-MS under the same conditions described in global metabolic profiling. The initial oven temperature was set at 70 °C and was programmed to increase to 300 °C (at 10 °C/min) and then to 320 °C (at 3 °C/min, hold 10 min). Calibration standards of anthricin were prepared in the range of 250–300 μg/mL and analyzed in triplicate. The peak area ratios (peak area of anthricin/peak area of internal standard) *vs.* concentrations were used for obtaining the calibration curve. The absolute concentration of anthricin was calculated from the regression equation of the calibration curve. The regression equation, *R*^2^ value, and absolute concentration of anthricin were represented in Table 3.

### 3.5. Statistical Analysis

The resulting data sets in the Microsoft Office Excel (version 2007; Microsoft, Redmond, WA, USA) were used for principal component analysis (PCA) and orthogonal partial least-squares discriminant analysis (OPLS-DA) by SIMCA-P software (version 13.0; Umetrics, Umea, Sweden). All spectra data were mean-centered and scaled with Pareto (par) scaling. To confirm the significant difference of each metabolite, the analysis of variance (ANOVA) test was performed using IBM SPSS Statistics 19 software (IBM, Somers, NY, USA), followed by the Tukey’s significant difference test.

### 3.6. Western Blot Analysis

HCT116 cells plated out into 60-mm plates at a density of 5 × 10^5^ cells/mL were incubated with CO leaf extracts. Cell were harvested, washed twice with ice-cold phosphate-buffered saline (PBS), and lysed in lysis buffer on ice for 25 min. The lysates were clarified by centrifugation at 14,000 rpm for 10 min at 4 °C. Protein concentrations were determined using the Bio-Rad protein assay kit (Thermo Scientific, Rockford, IL, USA) with bovine serum albumin (BSA) used as the standard. An equal amount of protein (35 μg) from each sample was separated using 12% sodium dodecyl sulfate-polyacrylamide gel electrophoresis (SDS-PAGE) and transferred to polyvinylidene difluoride (PVDF) membranes for 1.5 h. The membranes were blocked with 5% skim milk in tris-buffered saline (TBS)-Tween (0.1%) for 1 h at room temperature. Thereafter, the membranes were incubated overnight at 4 °C with a 1:1000 dilution of antibody. Primary antibodies used in this study included those against ERK, β-actin (Santa Cruz Biotechnology, Santa Cruz, CA, USA), p38, PARP, caspase-3 (Cell Signaling Technology, Danvers, MA, USA), JNK (BD Pharmingen, San Diego, CA, USA), and p-JNK (Invitrogen, Carlsbad, CA, USA). After washing three times with TBS-Tween for 5 min, the membranes were further incubated with a 1:5000 dilution of anti-mouse IgG or anti-rabbit IgG secondary antibody (Cell Signaling Technology, Danvers, MA, USA) for 1 h at room temperature. Subsequently, membranes were washed three times with TBS-Tween and incubated with enhanced chemiluminescence (ECL, Bionote, Hwaseong, Korea) reagent for 1 min, before the immunobands were visualized and quantified using Bio-Rad ChemiDoc and Bio-Rad Quantity One software (Bio-Rad Laboratories, Hercules, CA, USA). Image J software [52] (Windows version of NIH Image) http://rsb.info.nih.gov/nih-image/was used for charting the results of the western blot.

## 4. Conclusions

In summary, our results showed that the methanol extract of CO leaves had anti-proliferative activity against a human colorectal cancer cell line (HCT116). In addition, anthricin was suggested as the major contributing compound for the anti-proliferative activity, based on the results of GC-MS-based metabolic profiling of the CO leaf extracts. Anthricin was detected in the CO leaves, and JNK activation was found to be the mechanism underlying the apoptotic effect of this compound against HCT116 cells. Additional experiments including *in vivo* test in a suitable animal model are required to demonstrate the therapeutic effect of CO leaves. This is the first report on the anti-proliferative activity of CO leaf extracts against HCT116 cells, and these results suggest that the methanol extract and anthricin derived from the CO leaf could be used for the development of medicines possessing anti-colorectal cancer activity.
